# “It Doesn’t Feel Like You Can Win”: Young Women's Talk About Heterosexual Relationships

**DOI:** 10.1177/03616843221135571

**Published:** 2022-11-13

**Authors:** Tanja Samardzic, Paula C. Barata, Mavis Morton, Jeffery Yen

**Affiliations:** 1 Department of Psychology, 3653 University of Guelph, Guelph, ON, Canada; 2 Department of Sociology and Anthropology, 3653 University of Guelph, Guelph, ON, Canada

**Keywords:** heterosexual relationships, young women, silencing, communication, discourse analysis

## Abstract

Scholars have long explored the expectations of women to maintain intimate relationships and the gendered discourses governing those expectations. Despite the dating landscape changes, having intimate relationships remains important for young women. Amid these changes and the impacts of #MeToo/#TimesUp, investigating the discourses at play within women's talk about intimate relationships produces a current snapshot that contrasts with past literature. Young, heterosexual women of diverse racial, educational/work, and relationship backgrounds aged 18–24 years (*N* = 28) attended one of five online videoconferencing focus groups. Using an eclectic theoretical approach informed by feminist post-structuralism and discursive psychology, we analyzed women's talk about doing relationships. Mobilizing a discourse of intimate relationship necessity/importance, young women (a) were positioned as “the silenc(ed/ing) woman,” demonstrating a shared understanding of the necessity of silence when doing intimate relationships; and/or (b) actively took up “the communicative woman,” which they conceptualized as the hallmark of a healthy relationship. Tensions between these subject positions were evident (e.g., needing to be “cool”). Also, women described no-win situations in relationships despite attempts to contend with these contradictions and limitations. These findings may contribute to educational materials and youth programming delivered in high school or college.

Despite seeming liberation from the relational destinies that have governed girls’ and women's lives (e.g., Cairns, 2014; Harris, 2004), scholars argue that the importance of heterosexual relationship achievement and maintenance remains paramount (e.g., Abbott et al., 2021; Belenky et al., 1986; Brown & Gilligan, 1992; Jack, 1991; Ogolsky & Bowers, 2012; Tolman, 2002). Predicated on the understanding of this importance, we undertook an exploration of young women's talk about doing heterosexual relationships with men. By exploring young women's talk amid sociopolitical/cultural changes and advancements (e.g., #MeToo, #TimesUp), we aimed to compare and juxtapose how young women were talking about relationships today with foundational works (e.g., Belenky et al., 1986; Jack, 1991) that demonstrate that prioritizing one's partner and his needs at their own expense was the way to do heterosexual relationships.

## The Heterosexual Relationship Imperative

In adolescence and early adulthood especially, women in Western society feel they must be partnered to adhere to normative practices (e.g., Tolman, 2002). Chung (2005) has even described the dating relationship as an institution in which dating partners need to become indoctrinated in the engagement in and conformity to heterosexual practices. Young women are positioned by gendered discourses (i.e., structuring principles within society that are both constitutive of and reproduced in social institutions, modes of thought, and individual subjectivity; Weedon, 1987) in ways that conform to strongly heteronormative gendered stereotypes, scripts, and beliefs (e.g., “girlfriend”). These discourses are instrumental in reproducing systemic inequalities (e.g., Davis, 2018; Ken, 2008).

We have seen changes to the dating landscape (e.g., hookup culture; Bogle, 2008) and some challenges to the idea that women *need* to date and be in intimate relationships (e.g., the girl power discourse; Cairns, 2014). Such changes have occurred alongside a new post-feminist sensibility (Gill, 2007) that exhorts young women to take control of their own identity and exploit the unlimited possibilities available to them to achieve professional and financial success. Such opportunities challenge dated notions of needing to be partnered to be financially independent. There has been an updated construction of women as empowered, agentic beings who can freely choose their own femininity and have it all (Banet-Weiser et al., 2020 and Gill, 2007 offer descriptions and critiques). Gill (2007) has demonstrated how these ostensibly empowering subject positions are conditional upon and intertwined with women's participation in surveillance capitalism and self-marketing practices (Banet-Weiser et al., 2020). Discourses of femininity and heterosexuality both produce and constrain agency and shape what is seen as desirable or even possible concerning action within relationships (Gavey, 1989). Such constructions of empowered womanhood, especially since #MeToo/#TimesUp, are likely in tension with other constructions of womanhood associated with traditional femininity (e.g., selflessness). Relatedly, Bay-Cheng (2019) has suggested that agency, such as in the relationship context, has become a performative demand that young women must complete rather than a subject initiative they take up. The consequences of this performative agency include the obstruction of misogyny and its perpetuation of other forms of injustice (e.g., racism) and the expectation that young women perform in a “post-feminist masquerade” (McRobbie, 2008, p. 59).

## The Social Construction of the Ideal Girlfriend

Many traditional discourses available for young women to take up are predicated on the heterosexual relationship imperative (Ogolsky & Bowers, 2012). Scholars have recently demonstrated that three common discourses concern girls and women: discourses of feminine goodness, niceness, and silence. This non-exhaustive list provides a summary of the discourses at play that construct men's ideal girlfriend. A discourse of goodness entails selflessly prioritizing others' needs (e.g., Lafrance & Stoppard, 2006) and maintaining close connections with others (e.g., Tolman et al., 2016). This discourse positions women as moral and righteous and exists despite selflessness being an unattainable and self-defeating relational standard (Jack, 1991). Second is the niceness discourse. The imperative of women's niceness is grounded within accommodation, which results in the internalization of kindness and cooperation as desirable and acceptable behaviors (Rozee & Koss, 2001). For young women socialized with the idea that they need to be nice, not being nice can threaten their ability to hold onto relationships and ultimately threaten their self-identity (Schiller, 1997). Within the silence discourse, women's maintenance of intimate relationships rests upon their restricted expression of their true selves (i.e., being quiet; Jack, 1991). Silence is a gendered societal expectation and a necessary strategy to mitigate negative social effects (Parpart & Parashar, 2019), including the loss of a relationship (e.g., Abbott et al., 2021).

Women's behaviors are governed by discourses that associate silence and self-sacrifice with femininity and attractiveness (e.g., Abbott et al., 2021; Brown & Gilligan, 1992; Chung, 2005). These ubiquitous norms and standards, which largely reflect the “cultural universal” of White women (e.g., Fordham, 1993), persist. Ignored are the -isms and double standards inherent within such norms and standards. One example is the racism inherent in speaking up being “unladylike” among Black women but empowering among White women (e.g., Robinson et al., 2013). Contending with expectations to use one's voice thus creates a double bind for women. There is evidence, however, that some of these pertinent discourses have at least somewhat changed in recent decades (e.g., Cairns, 2014; Gill, 2007; Harris, 2004). Changes include how women date (e.g., finding a casual or committed partner on dating applications such as Tinder) and their dating choices (e.g., delaying or forgoing traditional domestic expectations; e.g., Lamont, 2013). It is thus likely that there are tensions between being empowered *and* nice, which necessitate young women grappling with these tensions (e.g., Banet-Weiser et al., 2020).

## Double Binds and Double Standards

Dominant discourses of gender and sexuality serve to perpetuate traditional constructions of cisgender male and female heterosexuality, and both highly influence women's heterosexual dating practices (Abbott et al., 2021). Also, many of the expectations that women in relationships be nice, good, and/or quiet put them in a no-win situation. For example, in the 1990s, women detailed avoiding conflict and maintaining their intimate relationships by restricting some aspect(s) of themselves from their partners (e.g., Jack, 1991). Self-sacrificing can then lead to the dissolution of the relationship given the lack of authenticity and unmet needs (Jack & Ali, 2010). Many of these expectations still, over two decades later, require that young women sacrifice some element(s) of themselves (e.g., needs, voice) to remain partnered (e.g., Abbott et al., 2021).

Feminists have illuminated the double binds and standards that exist for women. In heterosexual relationships, for example, male power is entrenched in dating rituals, such that men are the ones who are “supposed to” initiate and progress the relationship (e.g., assign the titles of boy/girlfriend; Chung, 2005). Despite such illumination efforts and coupled with arguments of women's increased agency in dating (e.g., Eaton & Rose, 2011), more recent research demonstrates that these gendered relations are continually upheld and reinforced. In Abbott et al.'s (2021) recent research, young people drew on discourses of heteronormativity and male dominance and ownership, where young women were positioned as quiet and laid back (e.g., not asking him to define the relationship despite wanting that reassurance). These participants upheld expectations that being silent is the way to *do* relationships. While recently there has been a renewed exploration into the concept of dating and some engagement with associated double standards/binds (e.g., Goldstein & Flicker, 2020), more research is needed to illustrate how young women discuss and navigate the expectations faced in heterosexual relationships. Specifically, more exploration is needed into the pressures to restrict parts of the self, especially in young adulthood when one's sense of self is developing.

## The Present Study

This study, which aligns with the goals of critical feminist research (e.g., Crawford & Marecek, 1989; Hare-Mustin & Marecek, 1988), follows two significant sociopolitical shifts: #MeToo, a global social media movement that illuminated the prevalence of sexual violence, and #TimesUp, which has aimed to end workplace sexual harassment. Both have drastically altered the sociopolitical climate and have provided more space for (young) women to use their voices more publicly (Gilligan, 2018). But despite these movements' impacts on awareness-raising, healing, and empowerment, there is evidence (e.g., Abbott et al., 2021) that young women are still silenced and silencing in efforts to maintain heterosexual intimate relationships despite changing gendered dating norms. This may be because these movements have focused less on long-term intimate relationships and more on sexual harassment and violence in other contexts. Thus, an investigation into the discourses at play in young women's lives at this moment in time as they come to age amid such important sociopolitical influences is crucial. This study was guided by two research questions: (a) What discourse(s) shape(s) the expectations that young, heterosexual, cisgender women have of intimate relationships? (b) How do young, heterosexual, cisgender women grapple with expectations about intimate relationships with men?

## Method

### Participants

Participants were 28 young women aged 18–24 years (*M* = 21.53, *SD* = 1.77). They all identified as heterosexual, cisgender females or women and were culturally diverse (61% White or European-Canadian, 36% racial/ethnic minorities, and 3% biracial/multiracial). Detailed demographic and relationship information by the participant can be found in Table 1.

**Table 1.** Demographic Information from Focus Group Participants.

FG	Participant Pseudonym	Age	Gender	Race/Ethnicity	Education	Employment	Presently a Student	Relationship Status	Number of Committed Relationships	Engagement in Sexual Activity
1	10	21	Female	White or European-Canadian	Some university education	Unemployed looking for work	Y	In a relationship	2	Y
	Charli	20	Female	Black or African Canadian or Caribbean-Canadian	Some university education	Employed part-time and student	Y	Single	0	N
	3	21	Woman	South Asian or South Asian-Canadian	Some university education	Employed part-time and student	Y	In a relationship	1	Y
	Jubilee	20	Woman	White or European-Canadian	Some university education	Employed part-time	Y	In a relationship	1	N
	Patricia Black	23	Female	Black or African Canadian or Caribbean-Canadian	Bachelor's degree	Unemployed looking for work	N	Single	0	Y
	Rebecca	22	Female	White or European-Canadian	Some university education	Employed part-time and student	Y	Single	2	Y
2	Ella	24	Female	White or European-Canadian	Some postgraduate education	Student	Y	In a relationship	2	Y
	Elizabeth	19	Female	Latin or Central or South American or Latin-Canadian	Some university education	Unemployed looking for work	Y	Single	1	Y
	Anna	19	Female	White or European-Canadian	Some university education	Student	Y	Single	0	Y
	Veja	24	Female	South Asian or South Asian-Canadian	Some university education	Student	Y	Casually dating	3	Y
	Gracie	20	female	White or European-Canadian	Some university education	Student	Y	In a relationship	1	Y
3	Four	21	Women	White or European-Canadian	Some university education	Unemployed looking for work and student	Y	Casually dating	1	Y
	Jenny	22	Female	East Asian or Pacific Islander or Asian-Canadian	Some university education	Student	Y	In a relationship	3	Y
	Alison	24	Female	White or European-Canadian	Bachelor's degree	Unemployed looking for work	N	In a relationship	2	Y
	Violet	19	Female	White or European-Canadian and Middle Eastern or Middle Eastern Canadian	Some college education	Student	Y	In a relationship	3	Y
	Burgundy	20	Woman	White or European-Canadian	Some university education	Employed full-time	Y	Casually dating	3	Y
	Christie C.	23	Female	White or European-Canadian	Bachelor's degree	Employed full-time	N	In a relationship	3	Y
4	Delilah	23	Female	White or European-Canadian	Some postgraduate education	Student	Y	In a relationship	2	Y
	Oshin	24	Female	East Asian or Pacific Islander or Asian-Canadian	Some university education	Student	Y	Married	3	Y
	Rachel	24	Female	White or European-Canadian	Some postgraduate education	Student	Y	Single	3	Y
	Catherine	23	Woman	White or European-Canadian	Bachelor's degree	Self-employed	Y	In a relationship	3	Y
	Sawyer	22	Female	White or European-Canadian	Some university education	Student	Y	In a relationship	2	Y
5	Ahbi	20	Female	South Asian or South Asian-Canadian	Some university education	Employed part-time and student	Y	Single	0	Y
	Molly	22	Female	White or European-Canadian	Bachelor's degree	Employed part-time	N	In a relationship	1	Y
	Carly	22	Female	White or European-Canadian	Some university education	Student	Y	In a relationship	1	Y
	Jessie	18	Female	East Asian or Pacific Islander or Asian-Canadian	High school diploma	Student	Y	In a relationship	1	Y
	Georgia	21	Female	West Asian or West Asian-Canadian	Some university education	Student	Y	Casually dating	3	Y
	Emma	22	Female	White or European-Canadian	College diploma	Employed full-time	N	In a relationship	3	Y

*Note.* FG = focus group; Y = yes; N = no.

### Procedure

After receiving university research ethics board approval, we recruited heterosexually identified women aged 18–24 years who studied and/or resided in Ontario, Canada to participate in one of five online videoconferencing focus groups from March to May 2021. We posted Facebook and Instagram advertisements and relied on word-of-mouth recruitment. We indicated that we were seeking participants for a focus group study to discuss topics like heterosexual dating, sex, and relationships in today's society. We also asked that participants either be alone or use headphones when participating to ensure privacy given the online nature of the study. We conducted this entire study online to comply with COVID-19 pandemic restrictions.

Interested participants emailed the first author (TS) for a link to the brief online survey, which 37 women completed. Of those, some did not reply to the participation invitation, one indicated that she wished to not continue after completing the check-in, and a few were unavailable at the selected focus group times. We planned to do several rounds of recruitment and prioritize participants based on diversity (e.g., once certain demographics were represented [such as White women], they would be excluded from further recruitment rounds). However, the participants in the first round were sufficiently diverse, and most of those who expressed an interest in participating were invited. TS conducted brief, individualized check-ins on Microsoft Teams before each focus group. This allowed her to explain the process of changing one's name to one's self-selected unique pseudonym to help maintain confidentiality. We required participants to log in as a guest, thus preventing those entering Teams in browser from seeing the entire group. Instead, those participants could only see the person who was speaking at the time.

TS conducted the focus groups on Teams, and an undergraduate research assistant was present to offer technical assistance if needed. Each focus group began with a reminder about our audio and videorecording of the focus groups and confidentiality, including its limits, and then proceeded to establish ground rules. Some groups selected to freely interject when wishing to speak, while others requested that they use the “raise hand” function in Teams, allowing TS to moderate the speaking order more carefully given concerns with participants who were in the focus group in browser mode. Before recording, they participated in an informal icebreaker activity where they said an interesting fact about themselves. Once recording began, TS oriented participants to the purpose of the focus group. Participants were instructed to think about other young women in the 18- to 24-year-old age range and were told that they were not required to discuss their own experiences (see Appendix A for the focus group interview guide). While participants were asked questions about their engagement in sexual activity, for this manuscript, we have focused only on participants’ discussions of non-sexual experiences. After the focus group ended, participants were sent an email with a community resource list and were either e-transferred $15 CAD or got a $15 CAD Amazon e-gift card.

### Theoretical Approaches and Analytic Strategy

Informed by feminist post-structuralism (e.g., Gavey, 1989) and discursive psychology (e.g., Potter & Wetherell, 1987; Wetherell & Potter, 1988), we analyzed young women's talk about how intimate relationships with men are done in today's society. To address the complexity of their talk, we chose an eclectic approach that allowed us to both (1) consider how broad regimes of knowledge and ways of speaking structure what is available for participants to say (feminist post-structuralism); and (2) attend to how participants' language use positioned them in specific ways (e.g., authentic or knowingly sophisticated; discursive psychology). From a feminist post-structuralist perspective, gendered subjectivities and heterosexual relationships are constructed in various discourses that are associated with regimes of knowledge about gender and relationships. Such discourses naturalize gender differences and heteronormative gender roles in relationships, obscuring their origins in patriarchal and colonial orderings of social reality (e.g., Weedon, 1987). Multiple discourses offer various subject positions, or possibilities for constituting subjectivities or understandings of the world (Weedon, 1987). Gavey (1989) argues that individuals do have some choice when positioning themselves in discourse, but this process results from a discursive battle for one's subjectivity.

A feminist post-structuralist approach to discourse analysis allows understanding of the contradictions and complexities inherent in people's views, positionalities, and experiences within the world (e.g., the same young woman may engage in competing discursive elements that conflict with one another—something we demonstrate below; see also Gavey, 1989). It was also important for us to say something about how our participants positioned themselves while constructing accounts of their relationships or when responding to others in their focus groups (Wetherell & Potter, 1988). Making claims about both was not possible in a strictly feminist post-structural discourse analysis. Furthermore, in inviting our participants to reflect on the contradictory positions afforded to them in relationships, we treated them as competent “analysts” of gendered discourse in their own lives. This slight divergence from a strictly constructionist stance in making claims about participants' lives outside of the focus group was, therefore, necessary to fully account for the nuances in their talk. We were interested in how the self was discussed and theorized and how participants constructed and conceived their version of events. What was important was the function that their representation of an account performed within the focus group context (Wetherell & Potter, 1988). We have thus thoughtfully and intentionally opted for a broader analysis of participants' talk, allowing greater flexibility in the claims we can make.

The focus group data covered multiple topics, so we focused on their talk about navigating intimate relationship expectations. TS transcribed the data verbatim via Express Scribe and manual transcript, then re-read the transcripts several times. TS and PB narrowed down the most prominent (e.g., references to traditional expectations of women) and most interesting (e.g., expectations to be communicative followed by condemnation for not being as such in relationships) elements within the extensive data excerpts that were reviewed. Next, TS (in consultation with PB and JY) conducted a more fine-grained analysis of the language used in these excerpts, attending to and qualitatively coding in NVivo 12 the details of the language that participants used and the function(s) that those linguistic choices served. This allowed us to attend to ways of speaking and assess the context and (dis)agreement between and within participants. All of the authors met twice (aside from TS's individual meetings and conversations with PB and JY) to discuss patterns in women's talk and methodological considerations. They also all provided written feedback on drafts of this manuscript.

### Reflexive Account

We have grappled with our positionality since the beginning of this project when TS first conceived her dissertation idea in close consultation with her advisor (PB), whose work is explicitly feminist and has centered around intimate partner violence and sexual assault. TS is a White, heterosexual, cisgender, first-generation Canadian woman whose adoption of a critical feminist approach to research on young women's expected practices was influenced by her own experiences, identity, and social location. The other authors serve on TS's dissertation committee, and each brings important elements to this work. MM's research interests in social justice issues and critical community-engaged scholarship and JY's expertise in discourse analysis and discursive psychology have resulted in slightly different readings of this manuscript, with valuable considerations that had not been previously considered being brought forth. We acknowledge that our cultural and class locations have shaped our focus on discursive dilemmas in this study that may be more familiar to White, cisgender, heterosexual, Western, middle-class women than to women occupying other, intersectional social locations.

## Results

We explored young women's talk within the context of an overarching discourse of *intimate relationship necessity* and the subject positions available to them. Young women mobilized this discourse in their talk, which was underscored by the importance of having intimate relationships with men. Within that discourse, young women (a) demonstrated a shared understanding of the necessity of *the silenc(ed/ing) woman* when doing intimate heterosexual relationships, an expectation that some participants repackaged in more modern ways; and (b) actively took up *the communicative woman*, conceptualizing communication as the hallmark of a healthy relationship, despite their talk reflecting ideals of silencing and the onus being on them to communicate correctly in the relationship. Finally, some described being *the bad bitch* as their way of contending with the contradictions and limitations within a discourse of intimate heterosexual relationship necessity. However, positioning as the bad bitch was a fundamental threat to the normative gendered practice of intimate relationship maintenance.

### Intimate Heterosexual Relationship Necessity

Participants' talk was predicated on the shared understanding that being in intimate relationships with men was both important and necessary. They understood the necessity of adhering to societal expectations of normative heterosexuality (e.g., following the traditional trajectory in heterosexual relationships) and mitigating the perceived negative effects of expectational transgression. The risks associated with a failure to secure an intimate relationship include perceptions as the “dreaded Other” (e.g., Fahs, 2011) and/or as defective (e.g., Addie & Brownlow, 2014), as well as feeling the discomfort associated with singlehood. When asked about this, participants emphasized the censure that results from not adhering to relational expectations:

Burgundy (20, White): I just came out of a two-year-long relationship. And since then, I've basically been in my head. I'm like, oh, no. What if I go back to school in September and [people] think that if I'm not in a relationship, that there's something wrong with me. So, I think it kind of scared me a bit in how important people take being in relationships and how people can value you differently based off whether you're in a relationship.

Four (21, White): yeah, I think when you're younger it's not as important. As you get older, people are like, oh, you’ve never had a boyfriend. That's so weird. There's something wrong with you. When are you going to get a boyfriend?

Their talk demonstrated the positioning of single women as “scary,” “weird,” and “wrong” yet without them necessarily agreeing with this construction. We saw women acknowledging the importance of having a boyfriend while simultaneously exempting themselves from that requirement, at least temporarily. Jessie (18, East Asian), for instance, demonstrated her modernness as a high-achieving woman while also highlighting the eventual importance of adhering to traditional practices: “at our age, I would say [what matters] is being a high achiever, so you should focus on school. Once you get older, you should probably start thinking about starting a family.” Inherent in their talk was the assumption that the natural progression of a young woman's life is finding a partner, likely for marriage and children (e.g., Russo, 1976). It was also conveyed that whether or not they delayed engagement in those practices now, they would need to eventually adhere.

Racialized participants discussed religious and cultural pressures that differed from those experienced by White participants:

Veja (24, South Asian): I'm not sure about you guys, but I think for me, there's a standard you have to fulfill in terms of your family and your cultural and religious expectations.

Elizabeth (19, Latin-Canadian): I agree, yeah. I feel like for me, it's from a Catholic perspective. There's a lot more pressure on women in terms of relationships. Religious families, like you were saying, there's a certain follow through and a life as a woman where it's like you meet someone, you're in a committed relationship, but you're not doing anything, you get married… That's it. Standards are very hard to play with and I feel like can even cause a lot of internal issues. Like, am I a bad person? Am I failing as a Catholic woman?

Veja: and for me in terms of dating multiple people, it's like the first time you get into a relationship, your family kind of expect that that's the one that's going to last and that's the person you're going to marry. But then when it goes through or you break up, they're like wait, what is going on? Is there something wrong with you? Why are you looking to date multiple people? But you grew up in a Westernized society where it's okay to see what your options are. A lot of families in my culture, it's like no dating while you're in school but then as soon as you graduate, it's “well, when are you getting married?”

The intersections of gender, culture, and religiosity created a double bind for women when it came to relationships. On the one hand, there was an attempted delay in the need to engage in the seemingly required practice of waiting before dating. Cultural and religious factors that required dating in the “right” way also existed. Their talk underscored the constraint put on them to not date more than one person, despite navigating relationships in a context that encourages “see[ing] what your options are,” as Veja said. Regarding dating early and dating around, badness and defectiveness were inherent features of participants' talk. Despite important differences between this excerpt and the previous one, both reflect contemporary versions (e.g., school and then a family; Gill, 2007) of the social pressures for marriage and reproduction, which are expectations that have existed and been placed upon women for centuries (e.g., Russo, 1976).

Some participants, however, questioned other young women's uptake of these expectations:

Patricia Black (23, Black): I feel like some women get into relationships to validate their beauty and that they're worthy, valued, and sought after. Right? That's why I feel like there's serial monogamy. Once somebody is no longer in a relationship, get in a new one.

Jubilee (20, White): I have a best friend like that. Since grade nine, she hasn't gone maybe a 4-day period without being in a relationship.

Rebecca (22, White): yeah, I have a friend like that. She stopped the other day talking to a guy and the next day, she's like, guess how many dates I have? I was like, what? That's crazy.

While ostensibly this excerpt suggests a passing of judgment on young women who are frequently in relationships, we understood this as a linguistic strategy used within the focus group to distance themselves from societal notions of relationship importance for fulfillment. By doing so, they positioned themselves as having surpassed and transcended these “crazy” requirements. But as we will demonstrate below, nonadherence to the expectations discussed by participants is understood as a transgression against traditional Western gender norms. We will also highlight that transgressing and having relationships were worked up as mutually exclusive.

#### Being Positioned as the Silenc(ed/ing) Woman

Participants demonstrated a shared recognition of the expectation that young women engage in silencing behaviors as a way of doing intimate heterosexual interactions. Some reflected upon the typical stereotypes of normative femininity (Chrisler, 2013), like Ahbi, a 20-year-old South Asian woman (“in my community specifically … you don’t get equated to being a good woman if you’re not able to take the baggage in silence”) and Elizabeth, a 19-year-old Latin-Canadian woman (“women aren’t expected to be outspoken and give their opinions”). Violet's (19, White/Middle Eastern) use of a personal example, saying that both she and others have “sacrificed [their] happiness and placed [it] last” to put their partner's needs first in intimate relationships, and Jenny's (18, East Asian) agreement (i.e., “if someone says to do something, you’re not supposed to object. You’re supposed to be complacent and follow orders”) solidify this shared understanding. Portrayals of what we term the ideal girlfriend, the one our participants (irrespective of previous experiences and backgrounds) alluded to as required for doing relationships “right,” were colored with the language of silence, restriction, and self-sacrifice. This reflects women's language in studies that occurred decades ago (e.g., Belenky et al., 1986; Jack, 1991). Phrases such as “hav[ing] to” (e.g., Veja) were clear examples of the extent to which silencing and being silenced were recognized and, as we will demonstrate, critiqued by participants as being an ingrained element of normative femininity.

Participants were aware of the need to not speak up, particularly early in the relationship, as a cornerstone of normative femininity (Jack & Ali, 2010):

Veja (24, South Asian): you just don’t speak up about certain things that are bothering you, especially in the beginning of a relationship. If something happened, it's nothing that serious and you're probably making something out of nothing, especially in the beginning because you don’t want to bring it up and be that girlfriend that's like, wow, why are you stressing me out already? We just started dating.

Ella (24, White): yeah, even going off of that, you’re expected to be cool, especially early on, you want to be cool with everything. You almost feel like you have to have that period where it's perfect, it's the honeymoon phase so that they don’t complain about you to their friends. You want to make sure you have that positive light about you when they talk about you and when he thinks about you.

Both young women understood the pressure to silence or self-suppress. This suggests that speaking up is a threat to how they are perceived and their success with relationship maintenance. Both spoke about the need to embody this “cool” girl persona, a mask skillfully worn in an attempt to maintain a relationship (Flynn, 2012). Veja's account reflected a questioning of what is (not) worth bringing up, while Ella invoked and named the cool girl. This positioning of themselves as knowing they must be cool (i.e., quiet) was also referenced by Burgundy (20, White) when speaking about the movie *Gone Girl*: “[she is] the girl who goes to bars with her boyfriend, eats wings, and hangs out with his friends. It's this persona that's kept on for a long time, at least in the girlfriend phase.” Simply, “cool” behaviors silenced women in traditional ways, but our participants evoked modern, progressive language within the focus group and thus positioned themselves as nontraditional (e.g., Ella's connection of coolness with silence). Burgundy's addition solidified the importance of maintaining coolness/silence, suggesting that it is needed to maintain girlfriend status. Their fears of *not* silencing, especially because of fears about what others think, were also clear:

Emma (22, White): I find women, in particular, always have to be nice [and] dance around crazy or the b-word. I find you’re always walking on eggshells when you’re talking to men in particular so that you are not called one of those two things… I think, you know, there's nothing worse than being called one of those as a woman.

The risk of being perceived as not nice, a bitch, and/or crazy was worrisome enough to keep young women adhering to societal expectations (Chesney-Lind & Irwin, 2008). Emma's account illuminated the power that men hold in heterosexual relationships to decide when their girlfriend is “too much.” Young women must therefore avoid being called crazy and/or a bitch, a position that they actively avoided given its pathologized nature.

The necessity of silence and adaptation to maintain a relationship permeated participants’ talk in powerful ways:

TS: what sorts of things do young women do in relationships to try to maintain that relationship?

Charli (20, Black): I think sometimes, if they have an issue or something that they’re not comfortable with, they don’t often bring it up because it might destroy your relationship.

Patricia Black (23, Black): I’ve seen this thing on Twitter that's like men will never forgive you, but women will always forgive because they think there's redemption and [all] that but if a woman does something wrong in a heterosexual relationship, the man wants to be like it's over, it's done. They’re not willing to compromise and work through it.

Rebecca (22, White): I’ve had a couple friends who have completely altered their values and lifestyle based on [intimate male partners].

Patricia Black: mhm, and it's like if you change these things about us, somehow, he’ll love you more.

3 (21, South Asian): yeah, going off what Patricia said, in my current relationship, in the first year, it's not even just my relationship but I’ve seen this with my friends as well. But we try to make the guy feel happy and then change. So, the girl's always changing.

This excerpt was notable for several reasons. First, despite questioning relational expectations, participants from different social locations shared an understanding that such expectations are intractable and ultimately unavoidable. Both Patricia Black and 3's use of “always” highlighted the ubiquitous nature of these expectations. Second, this exchange evidenced the expectations of young women to be silent, change, and forgive as they do relationships. Their talk about needing to change reflected the patriarchal and androcentric context in which they do relationships. In this context, their thoughts, preferences, and experiences are seen as irrelevant or inferior to those of men (Gilligan, 2018). The discussion about needing to forgive spoke to the ingrained gender norms implicated in normative femininity, where women are meant to be the caregivers, including in relationships (Gilligan, 1982). Especially noteworthy was the way that they positioned themselves as understanding the expectations of silence as a hallmark of doing intimate relationships with men. Still, their talk demonstrated their lack of eagerness to position themselves as silencing in the same way that they actively aligned themselves as communicative (demonstrated below).

#### Taking up the Communicative Woman Position

Several participants situated their talk about relationships with men within the conceptualization of a “healthy” relationship as one where both parties, but especially women, are communicative (Eaton & Rose, 2011). Despite evidence of women needing to restrict aspects of themselves (Jack, 1991), the young women (e.g., Carly, 22, White), noted the centrality of communication: “if people don’t communicate, it's duped because the communication is most important.” For Sawyer (22, White), goodness was synonymous with communication:

I think a good woman is someone who's able to stand up for themselves and any of their beliefs without feeling that their voice can’t be heard… I think having open communication is a good strategy and always check, not always, but frequently checking in on your partner's well-being so that you know how everyone's feeling.

Sawyer's reference to knowing how everyone is feeling demonstrated her identification with engagement in emotional labor. According to Hartley (2018), emotional labor describes unpaid, invisible work regarding the management of emotions and life that women often do to keep those around them satisfied. Her account, in addition to others’ accounts, demonstrated an invocation of the assertive communicative women. This was then easily adapted in the service of traditional discourses about maintaining relationships (e.g., checking in on the male partner).

Participants actively positioned themselves as communicative, which often included discussion about their partner also being communicative. One example is Rebecca (22, White): “I think a good relationship is based on communication so therefore, a good girlfriend should be able to communicate same as a good boyfriend should be able to communicate.” As evidenced below, the importance of communication for maintaining important intimate relationships was readily adopted and accepted:

TS: sometimes not speaking up when things are bothering you is expected of young women in relationships. Would you say that a good girlfriend is one that doesn’t speak up?

Rachel (24, White): I think my ultimate answer is no, I don’t think that's right because then you’re not… I think you need to bring it up because otherwise, it's going to turn into something bigger and become a monster in your relationship. It's more important how you bring it up and when you bring it up to not target that person as if they’re a horrible person and doing something wrong. It's more the approach you take to make sure that you are communicating well and effectively to make a change or to bring up the concern.

Sawyer (22, White): I think, yeah, you should speak up because it would just drive you apart further. And a good boyfriend would care that something's bothering you and would rather probably you communicate that instead of… I feel like they would feel a lot worse if they knew they’re doing something that hurt your feelings.

Delilah (23, White): I’m just going to be echoing what's already been said but I think it might end up actually making you a worse girlfriend overall if you don’t confront issues that are bothering you and let your partner know because you might grow to resent them and end up not liking them. You have to give them at least the benefit of the doubt that they’ll want to change or try to fix whatever's bothering you.

Here, participants highlighted the importance of communication as being integral to relationship maintenance. However, the responsibility to communicate properly (e.g., Rachel) fell on the young women, and the focus was on how he felt (e.g., Sawyer). Here, it was not just that being communicative was enough; it was also about doing it in the appropriate or correct ways.

Implicated in discussions of communication was the idea of healthy relationships, which was juxtaposed with the type of relationship discussed by women who described silencing above. In one focus group, the young women strategically used specific terms to position themselves as psychologically sophisticated and modern:

Burgundy (20, White): I feel like the general term for a good girlfriend, at least for me, is being kind of supportive towards the positive growth of another person. Of course, of whatever's positive for them. But you are just kind of looking out for what's best for them and not really completely focusing on what's best for yourself in that case too.

Alison (24, White): I think sometimes being a good girlfriend can be having that balance between spending time with your significant other but also each of you individually taking the time that you need alone to just focus on your own hobbies or friends.

Four (21, White): yeah, I would say being with someone and supporting them but letting them have their alone time. Letting them grow and definitely not being toxic for each other. Not feeding off of a toxic cycle because I’ve seen that type of relationship with a lot of my friends when they just are feeding off each other's negativity. So, definitely making each other feel good and supporting each other.

Terms like positive growth and balance are desirable given their equation with goodness. We interpreted their use of terms seen in popular culture (e.g., Gill, 2007) and in the academic literature on work/life balance and self-care (e.g., McNulty & Fincham, 2012) as their positioning of themselves as psychologically sophisticated. Doing so showed their transcendence beyond dated notions of feminine relationality (Jack & Ali, 2010). They also emphasized their modernity by suggesting that modern women can be supportive of their partners in a way that seemingly differs from the aforementioned 1980s/1990s conceptions of a good girlfriend. We interpreted their talk as an attempt to distinguish from more traditional conceptions of goodness by positioning themselves as empowered, outspoken, and well-informed. According to other young women (e.g., Debman et al., 2014), these are all necessary elements for a healthy relationship today. We also saw their talk as an attempted dismissal of traditional conceptions of a good girlfriend in favor of more modern relationship ideals. Despite their willingness to position themselves as communicative, they are constrained because their talk reverts to a concern for their partner and the relationship. In some ways, our participants repackaged traditional conceptions of the ideal girlfriend into more contemporary ways.

The degree to which responsibility for communication was accepted by some of our participants but not others may be a product of harmful cultural constructions that permeate public discourse. Examples include the “Angry Black Woman” stereotype (e.g., Walley-Jean, 2009), the “Strong Black Woman” schema (e.g., Abrams et al., 2014), and others (e.g., Pyke & Johnson, 2003) that necessitate and maintain women's silence. Jessie (18, East Asian) provided evidence for this:

In my culture, it's still really considered conservative. It's very traditional to what you should do as a woman. If I am just a little more outgoing and I speak a little too much, then that could be considered as a bad woman because I'm doing something that a typical woman shouldn't do. I should definitely express my feelings but not too much. Like, not going against the norms.

3 (21, South Asian) also discussed telling her boyfriend that she was “done with [his] ass” and feeling like she needed to forgive him for his behavior and Oshin (24, East Asian) referenced the importance of communication and honesty to maintain trust. We noticed a pattern of mostly White women (*n* = 10) positioning themselves as communicative compared to just three racialized women. This pattern contradicts previous researchers’ assertions that communication is a resource for racialized women to manage, resist, and/or undermine systems of power (e.g., Davis, 2018). This may be because they were in a more privileged position compared to others, and thus possibly able to more freely speak up.

### Silencing versus Communication

The young women’s talk demonstrated the tensions and clash between subject positions required for heterosexual relationships and newer discourses of authenticity and empowerment (e.g., Gonick et al., 2009). For instance, the participants’ talk highlighted this contentious contrast between communication and silencing:

Anna (19, White): it seems like there's a bit of a double standard, because sometimes [others] will be like, oh, well, you shouldn’t really talk to your boyfriend about any problems or how you’re feeling. But afterwards, if you break up, they’re like, well, did you try to talk to them about it? Was this the only solution? Communication is key. People always are like “communication is so important,” but then they’re also like, “you shouldn’t really talk about how you’re feeling in a relationship.”

Elizabeth (19, Latin-Canadian): I’ll tell people my ex did this, so I broke up with him. And they’re like why didn’t you try again? Why didn’t you give it one more chance? But it's like, why do I owe somebody one more chance? Why is it that when it's a man doing it to me, I’m the one that has to be like let's get to the root of it, let's work it out. If I did something like this to a man, all of a sudden I’m a bitch. I’m a crazy girlfriend. I feel like it's so common for men to dump women randomly, but when it's a woman, it's like talk to him. You need to persevere. You’re the feminine touch in the relationship, you can work through it.

Ella (24, White): I completely agree. Bumping off of that, I feel like being a good girlfriend, you’re the one expected to initiate those conversations. There's almost a stigma. Guys can’t just come and be like what's wrong? If you say what's wrong, it's like, your crazy girlfriend might pop off. It's almost like, why am I in the position where I have to bring this up every time? I’m not the therapist of this relationship.

Veja (24, South Asian): Ella, I agree 100%. Once I’ve said it to you the first time, you’re kind of like, okay, I feel like my feelings are extremely unvalidated, then I’m just not going to speak up anymore.

Ella: and that almost forces you to pick and choose what you want to have that conversation about. Then you’re like well, it's too early and I don’t want to always be the problem bringing stuff up and I have to be cool with them wanting to do stuff but at the same time, I don’t want to suppress my emotions.

In the same way that women in Muehlenhard and McCoy's (1991) study faced the double bind of openly acknowledging a desire for sex and facing negative sanctions (e.g., being labeled “easy” or refusing desired sex and being labeled “respectable”), our participants faced a double bind between being communicative and staying in relationships. But our participants also highlighted the unfairness of that bind through words like “why do I” and hearing from others that “you really should(n’t).” They also did this by providing examples of dialogue from others who had unreasonable expectations of them within the relationships (e.g., Anna's account). Additionally, they highlighted the contradiction in what was expected of them (e.g., communicating the right way) and indicated needing to do emotional labor (Hartley, 2018), something that the young women identified as socially shared. All of this was in addition to indicating the consequences of speaking out, with their discussion identifying them as being in a no-win situation.

Their talk about the contradictions that come with assuming the role of the ideal girlfriend also showcased their desire to assume the role of the ideal girlfriend in the “right” way. This element parallels works on friends-with-benefits relationships, where women discussed the need to not show too much emotion or get too attached to not threaten the relationship (e.g., Fahs & Munger, 2015). Next, the young women demonstrated having to teeter between assuming the role of the ideal girlfriend and the frustration inherent in assuming that role:

Alison (24, White): I’ve heard this kind of thing before with women who are in the beginning stages of dating and just getting to know men to date, you can’t be crazy, like, over the top. But also, it's like, you need to have a bit of drama within you to keep the interest. So, for a lot of people, probably it's a tough balancing act between not trying to overreact and be too much for someone but then not being too little and flatline for someone.

Violet (19, White and Middle Eastern): that's so true. I’m in a relationship and the guy that I’m seeing says I want you to be of those traits that we’re talking about. Things that women are often so beat down for. He's like, I want you to blow up my phone with notifications or I want you to be possessive. I feel like maybe if I was like that, that would be looked at as oh, she's crazy. She's psycho. I feel like a lot of guys say things like that and it turns around when women do act like that. And it's just very conflicting and difficult to navigate through because if I really did show up to this relationship blowing up his phone, I don’t know if him or his friends would think that was cool.

Jenny (22, East Asian): yeah, basically at the beginning of a relationship or seeing a guy, you’re supposed to not be clingy. You’re not supposed to be blowing up their phone. Some girls, and I have done it too, look at the time and be like I’ll wait 30 min to respond so I don’t look clingy. But then as the relationship goes on for longer, the guy's usually like why aren’t you clingy? It's like dude, what do you want from me?

It was not as straightforward as engaging in silence or talking about what is bothering them to foster a healthy relationship. Instead, women discussed that it was a bit of both; however, teetering between “clingy” and “cool” was difficult and frustrating (Hatfield & Rapson, 1995). But we see, especially with Violet, that there are risks associated with being clingy, even if the male partner openly requests it, given fears about his friends’ perceptions. We also see that being communicative is important, but only to an extent. There is a tension between being communicative and “dramatic,” especially at the beginning of a relationship, and not being too much too soon. Concurrently, engaging in the more traditionally silencing type behaviors and being more reserved can result in possibly being perceived as boring. Both reflect a no-win situation and threaten the ultimate goal of securing and maintaining intimate relationships. However, a few participants negotiated these contradictions by positioning themselves as what we term the “bad bitch.” This was a way of dismissing the importance of intimate relationships and the traditional expectations of normative femininity.

#### The Bad Bitch

In two focus groups, some identified with a position we term the “bad bitch.” This terminology is rooted in hip-hop feminism (e.g., Halliday & Payne, 2020) and can be found in the lyrics of performers like Ariana Grande and Megan Thee Stallion. Urban Dictionary (2022) users define a bad bitch as someone who is a “badass, solid chick with self-respect” and someone who is “confident, independent, and strives for herself.” Our participants’ talk reflected this:

TS: what do you think other young women in our current society would say a good woman is or acts like?

Patricia Black (23, Black): my personal standards of a good woman is just bad bitch energy. Like, what you’re giving off is that. I am the shit and I know that I am. I respect women so much for owning themselves and what the hell they are at the end of the day.

3 (21, South Asian): what Patricia said, in my culture, I try to be a bad bitch person but it's hard when it's kind of against the culture. Me and my friends, we’re pretty bad. We’re bad asses, literally bad asses. But when we’re with our families, it's a whole other story. We’re the outcasts because we’re behaving inappropriate[ly]. I don’t know how to say it because it's not inappropriate. I know whatever I do is not inappropriate, but they literally say, oh, it’s inappropriate. We’re not doing anything wrong. But it’s like, whatever culture you’re brought up in will really impact the way you view yourself. And it’s kind of hard when you have a culture that is completely different from the Western culture because here, you could wear what you want, do what you want, [and] say what you want. Even though it’s still restricted, you have more of a freedom. But I’m from a South Asian country so it’s very restricted, so even though you want to behave like a strong, independent woman, it’s so hard. The cultural differences literally pull you down when you’re even trying to be a strong individual in a country that's in a different place.

Patricia Black: I fully agree. I’m from an East African background and it’s so hard when you grow up in a Western space, and you have Western values intermingled with your cultural beliefs. Especially when your aunts and uncles are telling you to behave a certain way. And I find it so hard to shut my mouth [but] I don’t want to embarrass my parents. In my culture, people gossip and so I don’t want people to talk bad about my parents and be like, “oh, they raised the girl who doesn’t know how to shut up.” So I’m like, okay, I’m just gonna be quiet for this one moment and kind of subdue who I am and my true nature, just to appease them and then go back to how I am just so I don’t have to deal with it and they don’t have to deal with it.

The bad bitch was constructed by participants as representing the opposite of the woman who takes great care to adhere to how she should be in heterosexual relationships. The bad bitch does not concern herself with what society deems acceptable for young women and also actively rebels against that acceptability. This subject position draws on updated representations of women as strong, confident, and autonomous (Lazar, 2014) and echoes ideals of reclaiming femininity (Banet-Weiser et al., 2020). This is in direct contrast to more traditional conceptions of women as quiet and compliant, though we saw that these conceptions are indeed still applicable (e.g., 3's reminder that Western society is still restricted, though not as much when juxtaposed with the South Asian context).

But risks associated with positioning themselves as the bad bitch existed. We saw this with Patricia Black and 3's talk as the two women who invoked the bad bitch and their respective cultural constraints, which necessitated silence. Patricia Black's talk, especially, reflects Black girls’ inner struggles to both “pass” and enact their cultural identities as they struggle with being silent (e.g., Fordham, 1993). 3 and Patricia Black referenced doing this to maintain an appropriate image for their family's sake. The intersections of gender and culture were clear, especially when attempting to embody bad bitch characteristics amid tensions between their familial and Western cultural contexts.

We also saw further evidence of such risks regarding the bad bitch position:

TS: what does it mean to be a bad woman?

Ahbi (20, South Asian): I believe it means to be a badass woman. It’s making a man uncomfortable.

Carly (22, White): yeah, basically representing yourself and not listening to anyone; just kind of going by your own way. But I know a lot of girls who get asked out by boys, they say no, the guy keeps going and you’re seen as a bad woman by rejecting this person because apparently girls are supposed to love the chase.

Jessie (18, East Asian): yeah, I agree with what Carly says because, to my own experience and a lot of my friends’ experiences, it’s really hard for us to say no as a woman. If you keep on saying no again and again, then people will think there must be something wrong with you.

Emma (22, White): I like what most of us have already said is being a bad woman is saying no to a man, which I think is pretty gross.

Molly (22, White): yeah, as much as I don’t think anyone would explicitly go around saying you are a bad woman, I think if you don’t fit into a lot of those more traditional roles, people are probably thinking it.

Participants described themselves and others who identify with the bad bitch as badasses, autonomous, and strong-willed. On the one hand, participants described this way of doing relationships and emphasized the importance of embodying the aforementioned characteristics within the focus group. However, their talk about doing intimate relationships was tainted by risks (e.g., perceptions of defectivity or badness; e.g., Lafrance & Stoppard, 2006). These women live in a context where their actions and ways of being are constrained, so expressing (especially negative) emotions transgresses the way that women are expected to *do* gender (Chrisler, 2013). But the bad bitch herself was not constructed as negative by our participants, and some readily took on the subject position (e.g., Patricia Black [23, Black]: “I am the shit, and I know I am”).

Talk of the bad bitch was juxtaposed with talk of silencing, where the latter included a focus on other women as well as themselves at times. But this was done in the context of socio-cultural expectations (e.g., 3's talk of being seen as inappropriate for being a badass). Talk of the bad bitch was focused on their own actions and ambitions more generally, with a more positive connotation and a diluted focus on relationships amid participants’ discussions. But talk of silencing and/or being silenced elicited negative emotions that accompany self-sacrifice and giving up parts of oneself (e.g., anger). There are few opportunities for women to express anger and self-centeredness in socially sanctioned ways, with one exception being the premenstrual portion of a woman's menstrual cycle, which allows a sense of permission or legitimacy in expressing such emotions (e.g., Cosgrove & Riddle, 2003). Participants’ constructions and descriptions of the bad bitch may be another way that they feel they can transgress traditional relationship gender norms. This may be because although the bad bitch transgresses relational norms, she can be admired for her independence and adoption of masculine traits, which are valued in Western society. Alternatively, it may be that the bad bitch plays on modern characteristics (e.g., empowerment), which are admired and respected, particularly in some cultural spaces.

Though our analysis has shown that the positions available to young women within dating relationships often put them in lose-lose situations, we interpreted the bad bitch as their way of calling out of sorts, albeit paradoxically. The young women described taking up the bad bitch position as a way of doing relationships and contending with the contradictions and the limited options available to them within a discourse of intimate heterosexual relationship necessity. We also saw it as a way to demonstrate and normalize among the group that they do not care to engage in the performativity of silencing and/or communication. The bad bitch does not subscribe to traditional gendered expectations of women in relationships, nor does she care for the requirements of maintaining relationships more generally. For her, being true to herself matters, whether or not that allows for being partnered. However, as we have demonstrated, not adhering to gendered expectations and performing gendered behaviors in relationships threatens the ability to have and remain in heterosexual relationships. Maintaining relationships is something that most of our participants deemed important, despite attempting to distance themselves from that imperative.

## Discussion

We explored young women's talk about how to *do* heterosexual relationships in today's society, especially given the ever-changing social conditions that concern dating. Within a discourse of intimate heterosexual relationship necessity, young women described a shared understanding of being positioned as the silenc(ed/ing) woman and their willingness and desire to take up the communicative woman position. We saw evidence of tension between these two subject positions, such as with young women feeling the need to be the “cool” girl(friend). Coolness, when juxtaposed with more traditional relational behaviors (e.g., “shutting up”), was interpreted by us as being a more contemporary way of silencing within intimate relationships. However, positioning oneself as cool seemed to function in the same way as positioning oneself as quiet within a relationship. A few participants identified with the “bad bitch” subject position, which we saw as a paradoxical position used to manage the contradictory ways that heterosexual relationships are enacted. But positioning as such necessitated a willingness to reject intimate relationships altogether.

We saw evidence that young women were dismissive of dated notions of the traditionally good/ideal girlfriend as being acquiescent, quiet, overly caring, and fearful of relationship disillusion. These findings contrast earlier work such as Jack's (1991) findings of women's self-sacrificial over-caring and fears of relationship termination. Similar to recent findings (e.g., Lazar, 2014), we interpreted our participants’ talk as reflecting the characteristics of modern-day young women in relationships (e.g., as empowered and self-sufficient; Gill, 2007; Gonick et al., 2009; Lazar, 2014). But attempts at being a bad bitch were often condemned. This reflects Chesney-Lind and Irwin's (2008) assertion that all girls, regardless of class or race, are subjected to popular misogynistic narratives about their bad and destructive nature. Girls are monitored and their misbehaviors are continually identified and punished (Fordham, 1993; Harris, 2004). We assert that at their core, the discursive resources available to our participants, who, within-group, differed in important demographic characteristics, were similar to those available to the women in Jack's (1991) interviews. Despite contextual differences between Jack's (1991) participants and our own (e.g., the women in Jack's study were a majority White, American sample who were much older and often married), the binding link was the removal of parts of oneself for relational purposes (e.g., relationship security). Their talk allowed them to seem more progressive than the 1980s/1990s societal conception of a young woman and a girlfriend by portraying progressiveness and sophistication. But despite voicing desires to distance themselves from gendered relational expectations, the imperative for intimate relationships still permeated their talk.

Our participants appeared to dismiss dated notions of traditional femininity within the focus group context by positioning themselves as progressive and sophisticated. Their focus, instead, was on achieving personal growth and balance. This “post-feminist masquerade,” where women must balance masculine qualities of power with renewed pressures to perform normative femininity (McRobbie, 2008, p. 59), allows them to be seen as empowered and independent. This then devalues the importance of relationships. But as we demonstrated, relationships remain of utmost importance, as does the portrayal of today's ideal girlfriend as one who maintains the health of the relationship through compulsory communication and simultaneous adherence to traditional expectations of acquiescence and quietness (i.e., coolness). These findings are reminiscent of the girl power discourse, which emerged “…in the 1990s where girls *could* be active, [but] in the 2000s, they are now *expected/demanded* to be fully self-actualized … subjects” (Gonick et al., 2009, p. 2). The young women spoke about being cool and not worrying about creating conflict over little things, while simultaneously worrying that their boyfriends and their boyfriends’ friends would think they were “crazy bitches.” In the same way that scholars have questioned just how much power girls now have (e.g., Kearney, 2015), our results question the extent to which women can deviate from traditional standards in heterosexual relationships.

### Limitations and Future Directions

Our findings should be considered in light of some limitations. First, the focus groups were held online because of COVID-19 pandemic restrictions. This undoubtedly disrupted the flow of conversation given that most of the groups chose the “raise hand” function in Teams when speaking rather than a more natural approach employed in face-to-face conversations. Accordingly, there were multiple instances of young women changing the conversation topic, which may have prevented follow-up and reactions. Relatedly, although we asked that participants be alone or use headphones while participating, what they chose to discuss may have been constrained by the pandemic restrictions. Some participants had moved back in with loved ones or were living with roommates who may have been home during the sessions, especially since most focus groups occurred in the evening. Future researchers may find value in holding in-person focus groups to explore young women's talk about doing heterosexual relationships.

Another limitation was that our study design did not allow for a deeper exploration of important intersections (e.g., sexual orientation, cultural background) that impact young women's talk about doing relationships. We intentionally considered factors beyond gender as dimensions of subjectivity that have otherwise tended to be analytically inferior or less important (Bettie, 2000). While we limited our study to heterosexual women, it is important to explore discussions of doing intimate relationships with a more diverse sample concerning sexual identity to nuance understandings of societal pressure at the intersection of gender and sexual identity. Additionally, it is important to consider the way that other demographic characteristics impact those discussions.

### Practice Implications

These results may help frame discussions around dating and relationships for educational purposes, like in healthy relationship education programs or dating workshops delivered to youth in high school or college. Multiple programs aimed at teaching youth about healthy relationships exist, and several of those focus on or include information about dating violence prevention (Morton, 2019). What is missing is more critical thinking about why young women want intimate relationships so that they can recognize when the relationship is no longer serving them. Our findings may also help facilitate conversations with young women about the double binds they face when dating, especially with the increased use of online platforms. It may help to integrate some of our findings into educational material to help young women navigate the online dating atmosphere (e.g., texting and social media use while trying to avoid being perceived as “too eager” or “clingy”) and ensuring that their needs are met when initiating and developing heterosexual relationships.

## Appendix A

1. What are some societal expectations of young women today?
  - Where do those expectations come from?
2. What does it mean to be a “good” woman in this society?
3. What does it mean to be a “nice” woman?
  - What happens if girls are not nice? Are there consequences?
4. What does it mean to be “bad”?
5. How important is it for young women to be in an intimate relationship and to have a boyfriend?
  - Are there contradicting expectations for young women when it comes to having a boyfriend?
6. What makes a “good” girlfriend in an intimate relationship?
7. What sorts of things do young women do to maintain their intimate relationship with their boyfriend?
8. Sometimes, not speaking up when something is bothersome in a relationship is something that is expected of young women. Is a “good” girlfriend one that does not speak up?
9. How important is sexual activity (so anything from touching, oral sex, and/or penetrative sex) in an intimate relationship?
10. What are some expectations for young women when it comes to sex?
  - Are there contradicting expectations when it comes to sex, and if so, what are they?
11. Now that we have gone through the entire list of questions that I had prepared for today, is there something else that you think is relevant to mention given our discussion about dating and sex?

*Note.* We mainly focused on questions 1–6 in this study.
